# Molecular assays for the detection of microRNAs in prostate cancer

**DOI:** 10.1186/1476-4598-8-17

**Published:** 2009-03-06

**Authors:** Amara C Siva, Luke J Nelson, Chad L Fleischer, Mehrdad Majlessi, Michael M Becker, Robert L Vessella, Mark A Reynolds

**Affiliations:** 1 Gen-Probe Incorporated, San Diego, CA 92121, USA; 2 Department of Urology, University of Washington, Seattle, WA 98195, USA; 3 Puget Sound VA Health Care System, Seattle, WA, USA

## Abstract

**Background:**

MicroRNAs (miRNAs) are small non-coding RNAs (about 21 to 24 nucleotides in length) that effectively reduce the translation of their target mRNAs. Several studies have shown miRNAs to be differentially expressed in prostate cancer, many of which are found in fragile regions of chromosomes. Expression profiles of miRNAs can provide information to separate malignancies based upon stage, progression and prognosis. Here we describe research prototype assays that detect a number of miRNA sequences with high analytical sensitivity and specificity, including miR-21, miR-182, miR-221 and miR-222, which were identified through expression profiling experiments with prostate cancer specimens. The miRNAs were isolated, amplified and quantified using magnetic bead-based target capture and a modified form of Transcription-Mediated Amplification (TMA).

**Results:**

Analytical sensitivity and specificity were demonstrated in model system experiments using synthetic mature microRNAs or *in vitro *miRNA hairpin precursor transcripts. Research prototype assays for miR-21, miR-182, miR-221 and miR-222 provided analytical sensitivities ranging from 50 to 500 copies of target per reaction in sample transport medium. Specific capture and detection of mature miR-221 from complex samples was demonstrated in total RNA isolated from human prostate cancer cell lines and xenografts.

**Conclusion:**

Research prototype real-time TMA assays for microRNAs provide accurate and reproducible quantitation using 10 nanograms of input total RNA. These assays can also be used directly with tissue specimens, without the need for a preanalytic RNA isolation step, and thus provide a high-throughput method of microRNA profiling in clinical specimens.

## Background

MicroRNAs (miRNAs) are small non-coding RNAs of about 21 to 24 nucleotides in length and are derived from introns and exons of both protein-coding and non-coding genes. Many miRNAs are conserved in sequence between distantly related organisms and function in such essential processes as development, proliferation, differentiation, metabolic and signaling pathways, chromatin structure, and apoptosis [1-3]. MiRNAs can suppress translation of their target mRNAs via partial base pairing with the 3'UTR, generally requiring "seed" pairing of 6 to 8 nucleotides. Alternatively, in the case of perfect base complementarities, they can promote degradation of target mRNAs via the RISC complex in a process known as RNA interference. It is currently estimated that expression of over 50% of human protein-coding genes is mediated by miRNAs.

MiRNAs were first linked to cancer in 2002 when Calin et al. observed that miR-15a and miR-16-1 were down regulated in the majority of chronic lymphocytic leukemia patients [4]. Subsequent mapping of known sequences encoding miRNAs in the human genome revealed that greater than 50% of miRNA genes are located at fragile chromosomal sites, minimal regions of amplification or loss of heterozygosity, or common breakpoint regions [5,6]. Many of the miRNA genes residing in these fragile sites and cancer-associated genomic regions are arranged in clusters and similarly expressed, implying polycistronic primary transcription [5,7,8].

Expression profiling of miRNAs in cancer tissues has lead to discovery of miRNA "signatures" that are associated with tumor diagnosis and cancer-related staging, progression, prognosis and response to treatment [9,10]. MiRNA expression patterns have been shown to classify tumors by differentiation stage and tissue origin [11-13]. For diagnostic purposes, miRNAs could potentially be more directly associated with gene function than mRNAs, since they do not have to first be translated into proteins to have a biological effect. Furthermore, miRNAs may offer greater diagnostic sensitivity compared to proteins since they can be detected using quantitative amplification methods, including quantitative real-time PCR (qRT-PCR) and the real-time transcription-mediated amplification (TMA) methods described below.

Here we describe real-time TMA assays to detect miRNAs for potential oncology applications. For example, in the case of prostate cancer, prognostic markers that predict disease outcome may be more valuable than diagnostic markers that differentiate non-diseased tissue from tumor. Widespread serum PSA testing currently diagnoses both advanced prostate tumors and indolent cases; however indolent microscopic tumors do not require immediate treatment. Therefore, we selected candidate miRNA sequences that could potentially be used to discriminate normal tissue and/or indolent tumors from aggressive or metastatic disease. They were selected based on their location in or near fragile chromosomal sites that have been linked to prostate cancer progression and based on published reports of miRNA differential expression in prostate cancer tissues.

## Materials and methods

### Cell lines and xenografts

LNCaP, DU145, PC-3 and VCaP prostate cancer lines were obtained from the American Type Culture Collection (Manassas, Virginia). LNCaP and DU145 cell lines were cultured in RPMI Medium 1640 Custom (Invitrogen, Carlsbad, California) containing 10% FBS. The PC-3 cell line was cultured in MEM with Earle's salts (Invitrogen) supplemented with 10% FBS, 2 mM L-glutamine, and 0.9 mM sodium pyruvate. VCaP cells were cultured in DMEM containing 10% FBS. Freshly frozen samples of 21 human prostate cancer severe combined immunodeficient xenografts (LuCaPs 23.12, 23.1, 23.1AI, 35, 35V, 49, 70, 77, 78, 81, 86.2, 92, 93, 96, 96AI, 105, 115, 141, 145.1, 145.2, 153) were prepared using standard methods and supplied by R. L. Vessella. Characterization of several of these xenografts has been reported previously [14-16].

### RNA samples

Total RNA was extracted from cell lines using the Ambion mirVana miRNA isolation kit (Applied Biosystems Inc., Foster City, California) and frozen sections of xenograft tissue using Trizol reagent (Invitrogen) according to the manufacturer's instructions. Synthetic mature miRNAs were purchased from Integrated DNA Technologies (Coralville, Iowa). The human pre-miR-802 sequence was obtained from the miRBase [17] of the Sanger Institute and a synthetic hairpin precursor was synthesized using PCR amplification of the 94 nt pre-mir-802 sequence from LNCaP genomic DNA cloned into pBluescript II SK (+) and expressed as an *in vitro *transcript.

### Real-time quantitative PCR

cDNA was prepared from synthetic mature miRNAs or total RNA using specific TaqMan Assays-on-Demand reverse transcription primers and TaqMan miRNA Reverse Transcription Kit (Applied Biosystems Inc.). Absolute copy number of mature miRNAs was determined by qRT-PCR using TaqMan Assays-on-Demand primer and probe sets along with TaqMan Universal PCR master mix (Applied Biosystems Inc.) for cDNA amplification. Amplification and analysis were performed on the ABI 7000 sequence detection system. Copies per cell were determined from total nanograms of RNA using an estimated 15 picograms of total RNA per cell as described [18].

### Real-time transcription-mediated amplification

All buffer and enzyme reagents used in the real-time TMA assays were APTIMA^® ^reagents from Gen-Probe Incorporated (San Diego, California). All reactions were run in triplicate. Amplification reactions were prepared in 96-well microtiter plates containing specifically designed T7-provider [19] and 3'-extender oligonucleotides together with a common reverse primer and molecular beacon (see below). The plates were transferred to an Eppendorf Thermomixer R™ instrument and incubated at 42°C for 5 minutes. Next, APTIMA enzyme reagent was rapidly pipetted into each well and a sealing card was applied to the plate. After a brief mixing step (1 minute at 42°C), the plate was transferred to an MJ Chromo4 instrument (Bio-Rad, Hercules, California) that had been pre-heated to 42°C. Fluorescence readings were taken every 20 seconds at 42°C for 60 minutes. Emergence times were compared against calibration standards to derive miRNA copy numbers. The T7 provider oligonucleotides used in our model system experiments each contained a 3'-(reverse polarity)-dC nucleotide (5'-5'-phosphdiester linkage, designated "-Bl" in the sequences below) to block 3'-extension [19]. Real-time TMA assays used the following oligonucleotides for the different miRNAs: for miR-21, a T7-provider (AATTTAATACGACTCACTATAGGGAGAUAGCUUAUCAGA-Bl) and a 3'-extender (CGGTCGCAGAGATTAACTGGTACAGGGTTAAGCGTGGTCGACCGTCAACATCAGT); for miR-34b, a T7-provider (AATTTAATACGACTCACTATAGGGAGATAGGCAGTGTCA-B1) and a 3'-extender (CGGTCGCAGAGATTAACTGGTACAGGGTTAAGCGTGGTCGACCGCAATCAGCTAAT); for miR-182, a T7-provider (AATTTAATACGACTCACTATAGGGAGATTTGGCAATGGT-Bl) and a 3'-extender (CGGTCGCAGAGATTAACTGGTACAGGGTTAAGCGTGGTCGACCGAGTGTGAGTTCT); for miR-221, a T7-provider (AATTTAATACGACTCACTATAGGGAGACCACAACGGTTTAGCUACAUUGUCUG-Bl) and a 3'-extender (CGGTCGCAGAGATTAACTGGTACAGGGTTAAGCGTGGTCGACCGGAAACCCAGCAG); for miR-222, a T7-provider (AATTTAATACGACTCACTATAGGGAGAAGCUACAUCUGG-Bl) and a 3'-extender (CGGTCGCAGAGATTAACTGGTACAGGGTTAAGCGTGGTCGACCGGAGACCCAGTAG); and for miR-802, a T7-provider (AATTTAATACGACTCACTATAGGGAGA-CAGTAACAAAGA-Bl) and a 3'-extender (CGGTCGCAGAGATTAACTGGTACAGGGTTAAGCGTGGTCGACCGACAAGGATGAAT). All assays used the same reverse primer (CGGUCGCAGAGATTAACT) and molecular beacon labeled at the 5' end with FAM and at the 3' end with Dabcyl (CCGACAAGCGUGGUCGACGUCGG).

### Specific target capture of miRNAs

Capture of mature miRNAs was performed using the following chimeric hairpin target capture oligonucleotides (TCO) [20]: for miR-21 (TTTTTTTTTTTTUCAACAUCAGUCUGAUAAGCUAAAAAAAAAAAAA), for miR-182 (TTTTTTTTTTTTAGUGUGAGUUCUACCAUUGCCAAAAAAAAAAAAAAA), for miR-221 (TTTTTTTTTTTTGAAACCCAGCAGACAAUGUAGCUAAAAAAAAAAAA), for miR-222 (TTTTTTTTTTTTACCCAGUAGCCAGAUGUAGCUAAAAAAAAAAAA), and for miR-802 (TTTTTTTTTTTTACAAGGAUGAAUCUUUGUUACUGAAAAAAAAAAAA). The TCO was added with APTIMA Target Capture Reagent to RNA samples in Solution Transport Medium (Gen-Probe Inc.) and heated to 75°C for 15 minutes in a 96-well deep well heater (model IC25 with block 620–5036, Torrey Pines Scientific Inc., San Marcos, California) to denature the hairpin. The reaction mixture was then cooled to room temperature over 30 minutes to anneal the specific miRNA to the TCO. After binding to the miRNA, the 3' dA_12 _of the TCO was captured onto poly-dT_14 _derivatized magnetic beads (APTIMA Target Capture Reagent). The beads were collected using a Kingfisher96 PCR tip head (Thermo Scientific, Waltham, Massachusetts), washed in APTIMA wash solution, and mixed with APTIMA amplification reagent containing amplification and detection oligonucleotides in a 96-well PCR plate as described above. To liberate the miRNA from the TCO, the PCR plate was incubated at 90°C for 5 minutes and then immediately cooled on ice for 5 minutes.

## Results

### Design of TMA assays for the detection of miRNAs

**Figure 1:**
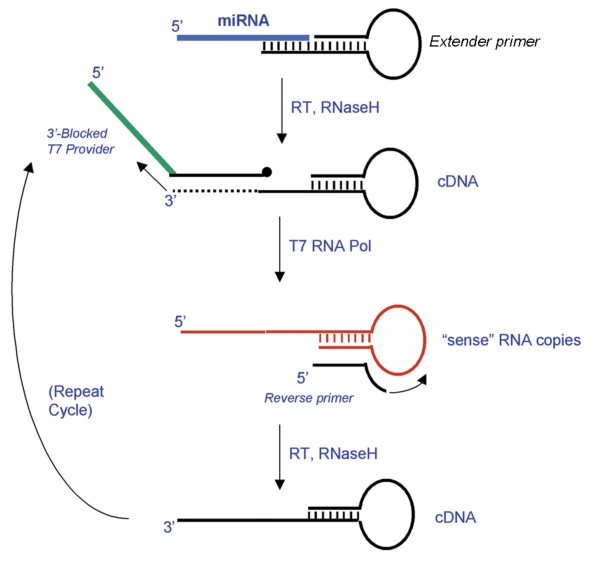
**Schematic of miRNA real-time TMA reaction**.

The amplification and detection of miRNAs is challenging due to their short length (approximately 21 to 24 nucleotides), thus restricting the possible oligonucleotides that may be used as amplification primers and detection probes. Previous PCR amplification methods have addressed this challenge through a variety of technical approaches, including the incorporation of locked nucleic acid modified nucleotide monomers for enhanced binding affinity to short sequences and the use of tailed or hairpin primers to increase the length of the resulting amplicons [18,21,22]. In the assays described here [19], an extender hairpin oligonucleotide first hybridizes to the 3' end of the miRNA. Upon addition of APTIMA enzyme reagent, the extender primer is extended to make a cDNA complementary to the miRNA and the RNaseH activity of the enzyme degrades the miRNA. A T7 provider oligonucleotide then binds to the cDNA and is extended. The 5' end of the T7 provider contains a promoter sequence where T7 RNA polymerase then binds and produces multiple copies of sense RNA that include the original miRNA sequence and an appended sequence that can be detected using a molecular beacon or other detection probe. Lastly, a reverse primer binds to the 3' end of the sense RNA copy and is extended to make another antisense cDNA template, thus allowing amplification of the sense RNA to be repeated in a cyclic manner (Figure 1).

### Real-Time TMA assays distinguish mature miRNA sequences

**Figure 2:**
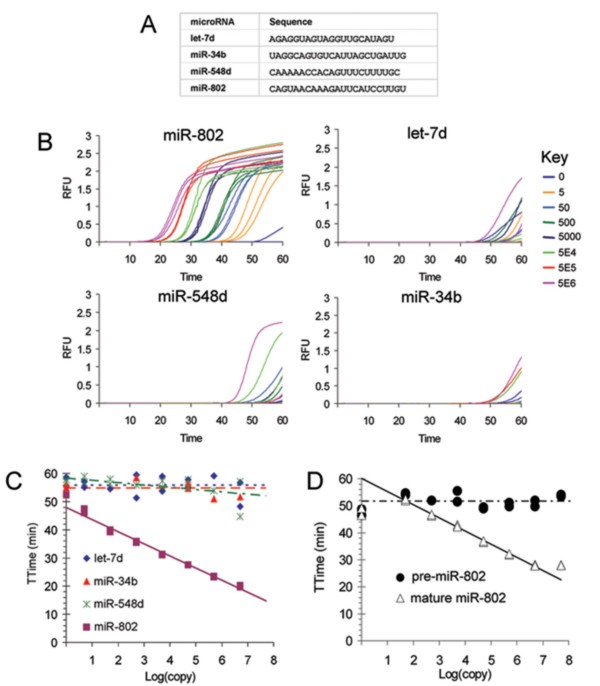
**Specificity of miR-802 real-time TMA assay**. A) Sequences of mature miRNAs tested. B) Amplification curves from tests of the miR-802 assay on various input copy numbers of synthetic miRNA targets. RFU: Relative fluorescence units. C) Calibration chart for the amplification curves in B. D) Calibration chart for the miR-802 assay tested on various copy number input of mature target vs. pre-miR-802 transcript.

The first target sequence for miRNA assay development was derived from searches in the regions of TMPRSS2 and Ets family member chromosomal breakpoints (21q21.2–21.3) because this region has been associated with aggressive forms of prostate cancer [23,24]. We selected miR-802, which resides in chromosomal region 21q22.12, due to its proximity to the TMPRSS2:ERG gene fusion. Our real-time TMA assay for miR-802 assay was shown to be specific for the synthetic mature miR-802 template when compared to unrelated miRNAs let-7d, miR-34b and miR-548d (Figure 2A, B, and Figure 2C). Some nonspecific amplification was observed for the let-7d and miR-34b targets after a threshold time (TTime) of 50 minutes (Figure 2B) and in control reactions that contained no template (data not shown). This model system demonstrated that 5 copies per reaction of this particular intended target, miR-802, could be detected above nonspecific background signals.

The specificity of the miR-802 assay for mature sequences was tested on a hairpin precursor in vitro transcript since mature processed miRNAs have different cellular functions from their hairpin precursors. As shown in Figure 2D, the miR-802 assay showed good discrimination of the mature miR-802 template from its corresponding hairpin precursor.

**Figure 3:**
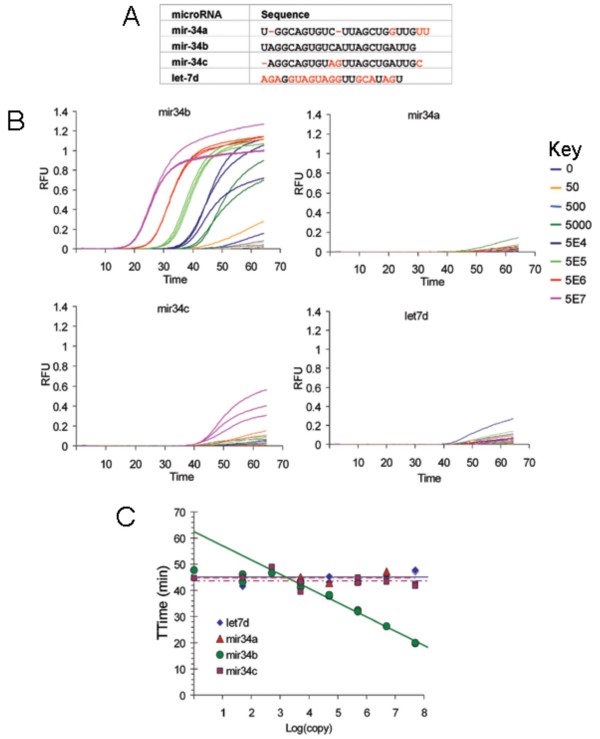
**Specificity of miR-34b real-time TMA assay**. A) Sequences of mature miRNAs tested. B) Amplification curves from tests of the miR-34b assay on various copy number input of miR-34 family members along with unrelated let-7d. RFU: Relative fluorescence units. C) Calibration chart for the amplification curves in B.

Since miRNA family members can differ in sequence by 1 or more nucleotides, we next designed a model system to demonstrate the sequence specificity of our real-time TMA assay. A real-time TMA assay was designed to specifically detect miR-34b and was tested against miR-34 family members and other non-complementary miRNAs. As shown in Figure 3, the miR-34b assay showed template-dependent amplification at about 20 minutes with 5 × 10^7 ^input copies per reaction using miR-34b synthetic miRNA template compared to synthetic mature miRNA family members miR-34a and miR-34c and the unrelated miRNA let-7d. Some cross-reactivity was seen with 5 × 10^7 ^copies per reaction of the related species miR-34c at about 42 minutes (Figure 3B), which is approximately the time that background signal was detected in the no template control reaction (about 45 minutes). Thus, the analytical sensitivity of this assay for the miR-34b species was less than 50,000 copies per reaction.

### Identification of miR-21, miR-182, miR-221 and miR-222 targets for assay development

**Figure 4:**
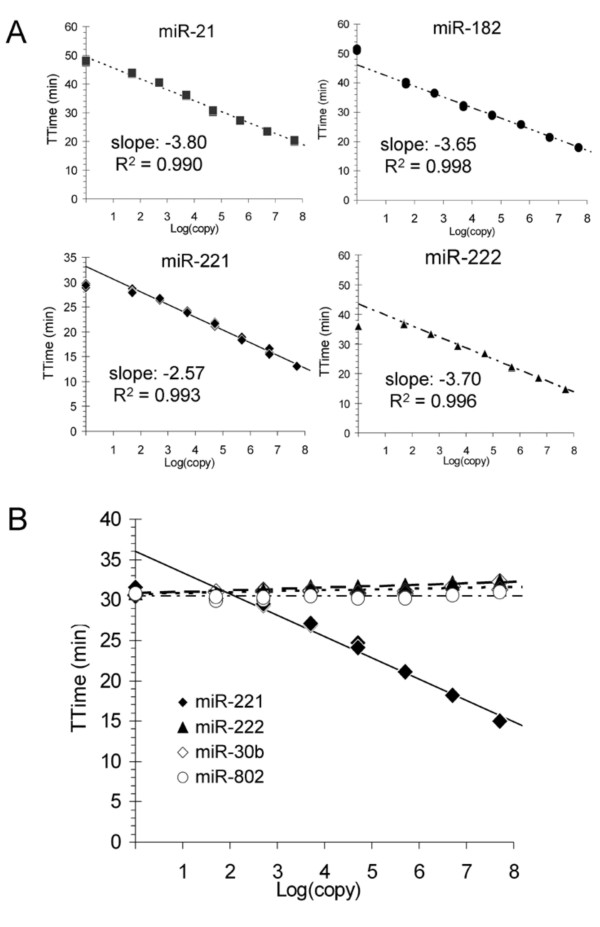
**Analytical performance of miR-21, miR-182, miR-221 and miR-222 real-time TMA assays**. A) Template-dependent amplification using the respective synthetic RNA miRNA targets. Amplification reactions were run in triplicate (without target capture). The slope and R^2 ^values were determined from log copy points 1.7 to 7.7 (50 to 5 × 10^7 ^copies per reaction). B) Specificity of the miR-221 assay using the corresponding synthetic miR-221 target as compared to synthetic miR-222, miR-30b, and miR-802 targets.

As mentioned in the Introduction, we prioritized several miRNAs based on their chromosomal location, putative mRNA target(s), and published microarray results that show differential expression between normal and cancer tissues. We also examined miRNA profiles of human prostate cancer xenografts to determine which miRNAs were differentially expressed compared to normal prostate tissue (see Additional file 1, Additional file 2, and Additional file 3). Based on this analysis, we concluded that miR-802 was not highly expressed in the xenografts and was not differentially expressed between normal and tumor samples. In contrast, we identified several miRNAs that showed high levels of expression and/or were differentially expressed in the xenografts. Four of these miRNAs, miR-221, miR-222, miR-21 and miR-182, were selected for further study based on the known biological functions of their predicted mRNA targets [25-32]. Real-time TMA assays were developed for each of these miRNAs after confirming their differential expression using commercial RT-PCR assays (see Additional file 4). As shown in Figure 4A, our real-time TMA assays for miR-221, miR-222, miR-21 and miR-182 showed good analytical performance. The specificity of our miR-221 assay was demonstrated using other synthetic mature miRNAs as targets (Figure 4B), analogous to our previous demonstration for the miR-802 assay (previous section).

### miRNA copy number determination in prostate cancer cell lines and xenografts

**Figure 5:**
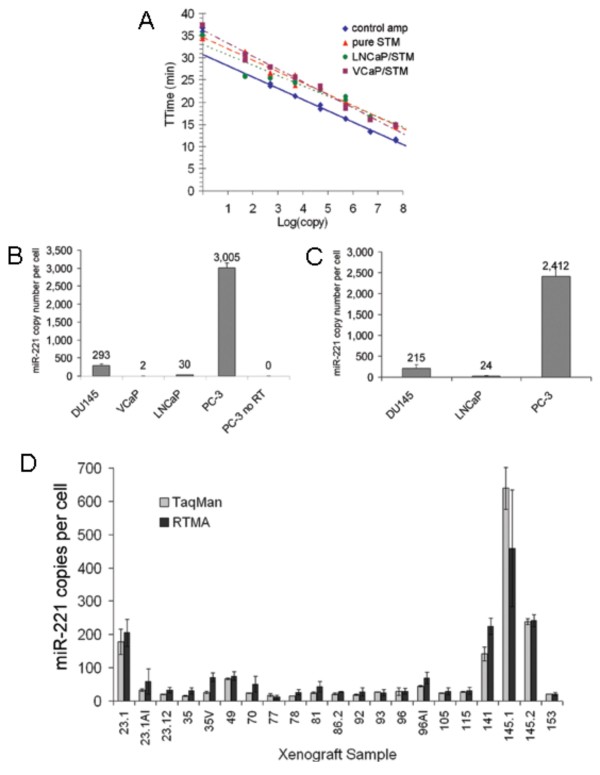
**Real-Time TMA miRNA assays with integrated target capture**. A) Capture and detection of known inputs of synthetic miR-221 added directly to STM buffer or to STM containing 10 ng of total cellular RNA (derived from VCaP or LNCaP cells, as indicated). Control reactions were run with synthetic miR-221 spiked directly into amplification reagent (without target capture). B) miR-221 copy levels determined by a commercial TaqMan RT-qPCR assay. C) miR-221 copy levels determined by our real-time TMA assay (with integrated target capture and amplification/detection). D) miR-221 copy numbers determined in prostate cancer xenografts using a commercial TaqMan RT-qPCR assay and also our research prototype real-time TMA assay (again incorporating target capture). For all real-time TMA samples, 10 ng total RNA was assayed per reaction in triplicate.

Previously, miR-221 was shown to have high expression in the PC-3 prostate cancer cell line but low expression in the LNCaP cell line [25]. To assess the efficiency of our real-time TMA assay for capturing and detecting miRNA from a complex mixture, known quantities of synthetic miR-221 were added to either Solution Transport Medium (STM) or STM containing total RNA extracted from LNCaP or VCaP cells (10 ng total RNA per reaction), both of which were shown separately to contain low or undetectable levels of endogenous miR-221 (see below). Control reactions were run with synthetic miR-221 transcripts added directly to amplification reagent (without target capture). Capture and amplification efficiencies for known input copy numbers of synthetic miR-221 were similar in the presence or absence of total cellular RNA (Figure 5A, curves for "LNCaP/STM" and "VCaP/STM" compared to "pure STM"). However, the assays that included target capture amplified somewhat less efficiently (about 10 to 20 fold) compared to the control reactions without target capture (Figure 5A, "control amp").

Endogenous expression levels of miR-221 were measured in the prostate cancer cell lines LNCaP, PC-3, DU145 and VCaP using a commercial TaqMan qRT-PCR assay (Figure 5B). We measured relatively low or undetectable levels of endogenous miR-221 in LNCaP and VCaP cells as reported above for our previous experiment (Figure 5A). Results from the TaqMan qRT-PCR assays showed about 3000 copies of miR-221 per PC-3 cell and about 290 copies per DU145 cell. Similar quantitation values were determined using our research prototype miR-221 real-time TMA assay, demonstrating analytical equivalence between the methods (Figure 5C). These tests were also performed on total RNA isolated from prostate cancer xenograft samples, again with similar quantitation values between the 2 assay methods (Figure 5D).

## Discussion

MiRNAs may be effective biomarkers for use in clinical diagnostics because they are extremely stable in body fluids taken from patients and formalin-fixed paraffin-embedded (FFPE) tissue sections, presumably due to their small size and protection by the RISC complex [33-38]. One advantage of the technology is that FFPE sections, in addition to fresh or frozen tissue, can be placed directly in STM for convenient transport and/or storage. The miRNA targets can then be specifically captured directly from the STM buffer without the need for additional RNA purification.

Although our research prototype miRNA assays performed well for some sequences, other sequences showed decreased amplification efficiencies, dependent on their length and base content. For example, we were unable to design an assay with acceptable sensitivity for one miRNA containing a high percentage of A/U bases in the 3'-extender binding region (data not shown). Increasing the length of the 3'-extender resulted in elevated background signals, presumably due to increased sequence overlap with the T7-provider oligonucleotide (ie resulting in non-specific amplification). Similarly, the need to discriminate between closely related family members presented constraints on the binding regions to which the 3'-extenders and T7-providers were designed, resulting in a trade-off between assay sensitivity and assay specificity. New assay chemistries are being investigated to mitigate these limitations. Based on the model systems described here, our real-time TMA assay appears to be best suited for samples that contain at least 5000 copies of the desired target miRNA per reaction.

According to our data, the miRNAs miR-221, miR-222, miR-182, and miR-21 were expressed at relatively high levels in prostate cancer xenograft specimens. Therefore, our research prototype real-time TMA assays for these miRNAs showed accurate and reproducible quantitation using 10 ng of total RNA from these tissues. Similar results were also obtained when these miRNAs were detected directly from STM extracted clinical FFPE specimens and urine sediments (data not shown). We are currently investigating endogenous normalization methods to provide relative comparisons of miRNA levels between specimens. Several miRNAs have been suggested for normalization purposes [39].

Based on the successful results, we suggest that real-time TMA assays could be effective tools for detecting miRNAs that exhibit moderate to high expression in diseased tissues. For example, miR-21 is reported to be commonly overexpressed in solid tumors of the lung, breast, stomach, prostate, colon, brain, head and neck, esophagus and pancreas compared to normal tissues. Several recent studies report that miR-21 downregulates four individual tumor suppressors: phosphatase and tensin homolog; tropomyosin 1; programmed cell death 4; and maspin. By targeting multiple tumor and metastasis suppressor genes, miR-21 has a presumed role not only in tumor growth but also in invasion and tumor metastasis [27-31]. Our results for miR-21 expression in tumor xenograft samples showed about a 5 to 25 fold upregulation in these tissues compared to normal samples with copy numbers ranging from 5000 to 35,000 copies per cell (see Additional file 4). Due to its implied role in cancer and its high level of expression in prostate tumor compared to normal prostate samples shown by our group and others [10,16,38,40], miR-21 may have value in distinguishing tumor from normal tissue.

Our results also showed miR-182 was highly upregulated in the majority of tumor tissues compared to normal samples (see Additional file 4). Calin et al. described the location of miR-182 in a minimal deleted region that is associated with an aggressive prostate cancer histotype [5] and others have also observed upregulation of miR-182 in prostate tumors [40,41].

Our real-time TMA assays for the tandemly expressed miR-221 and miR-222 could also prove to have clinical utility upon further investigation. These miRNAs impair apoptosis by targeting the tumor suppressor p27 (Kip1) [25,26,42]. A recent report has linked decreased p27 expression with an aggressive prostate cancer phenotype [43]. Interestingly, the miR-221 cluster is downregulated by androgen treatment [41], which may explain why miR-221 and miR-222 were downregulated in the results shown with the majority of prostate tumor samples tested here (see Additional file 4) and by others [16,41]. MiR-221 and miR-222 are overexpressed in PC-3 cells that exhibit an androgen-independent, highly metastatic phenotype. In contrast, miR-221 and miR-222 are expressed at relatively low levels in LNCaP and 22R*v*1 cells that exhibit slow-growing, non-metastatic phenotypes. We did not observe a significant difference in miR-221 or miR-222 expression in the three matched pairs of androgen dependent and androgen independent xenograft lines (LuCaP 23.1/23.1 AI, 35/35V, and 96/96AI, see Additional file 4); however, reported expression levels for miR-221 and miR-222 in patient biopsy cores, metastatic lymph node specimens, and non-malignant prostate tissues suggest that these miRNAs could be associated with invasive prostate cancer [25,40]. Based on these and other observations, miR-221 and miR-222 appear to be promising biomarker candidates for discriminating metastatic subtypes.

Clearly, clinical studies with large numbers of specimens would be needed to demonstrate the clinical value of the research prototype assays and miRNA biomarkers. Furthermore, it is possible that a panel of miRNAs would be required, assayed either in parallel or in multiplex formats, to provide sufficient correlation with disease outcome for prognostic applications. Considering the regulatory role that miRNAs play in normal and disease processes, it has been hypothesized that about 2 to 6 miRNAs could provide sufficient correlation for prostate and other cancers. Again, further clinical testing will be needed to confirm this hypothesis.

## Competing interests

The authors declare that they have no competing interests.

## Authors' contributions

AS participated in the design and coordination of the study, contributed to assay designs and methods development, conducted quantitative real-time TMA studies and data analysis, and drafted the manuscript. MR directed the design and coordination of the study, contributed to assay designs and methods development, and contributed to drafting the manuscript. LN and CF contributed to assay designs and methods development, and conducted quantitative real-time TMA studies and data analysis. MM and MB contributed to assay designs, and MM provided custom oligonucleotides for quantitative real-time TMA studies. BV provided the human prostate cancer xenograft specimens and assisted with the design of microRNA expression profiling experiments.

## Supplementary Material

Additional file 1 **Supporting information for miRNA expression profiling experiments**. Experimental methods used for microarray expression profiling of miRNAs extracted from human adjacent-normal and prostate cancer xenograft tissues. Differential expression of lead candidate miRNAs was confirmed using commercial quantitative RT-PCR assays (Applied Biosystems, Foster City, CA, USA). Click here for file

Additional file 2 **Key for specimens used in miRNA expression profiling experiments**. Descriptions for human adjacent-normal tissues (from prostatectomy specimens) and for human prostate tumor xenograft tissues (provided by Bob Vessella). Click here for file

Additional file 3 **Normalized miRNA expression values used for data analysis**. Data outputs from microarray expression profiling experiments (conducted as described in Additional file 1), with normalized values used in determining differential expression ratios between adjacent-normal and tumor xenograft specimens. Click here for file

Additional file 4 **Validation of miR-21, miR-182, miR-221 and miR-222 expression levels in human adjacent-normal and prostate tumor xenograft tissues using a commercial quantitative RT-PCR assay**. Bar graphs of copy numbers per cell determined using a commercial quantitative RT-PCR assay. Synthetic miRNAs were diluted 10-fold serially from 109 copies per reaction for construction of each standard curve. For each reverse transcription reaction, 10 ng of total RNA was used and amplification reactions were run in triplicate (see Additional file 1 for details). Click here for file
