# Supplementary material for: Molecular assays for the detection of microRNAs in prostate cancer
Source: Mol Cancer. 2009 Mar 6;8:17. doi: 10.1186/1476-4598-8-17 (PMC2656451; doi:10.1186/1476-4598-8-17)
Supplement: Additional file 1 — Supporting information for miRNA expression profiling experiments. Experimental methods used for microarray expression profiling of miRNAs extracted from human adjacent-normal and prostate cancer xenograft tissues. Differential expression of lead candidate miRNAs was confirmed using commercial quantitative RT-PCR assays (Applied Biosystems, Foster City, CA, USA). [file 1476-4598-8-17-S1.doc]

**Siva, A.C. et al. Supporting information**

**Supporting information text**

**Normal prostate tissue samples.** Five adjacent-normal prostate tissues, pathologically determined to be 100% normal (OCT-embedded), were purchased from Cytomyx Inc. (Lexington, Massachusetts). These samples were shipped to Asuragen Services (Austin, Texas) for subsequent RNA extraction and microRNA profiling.

**MicroRNA profiling.** Total RNA was extracted from 22 human prostate cancer xenografts using Trizol Reagent (Invitrogen, Carlsbad, California) with homogenization by Polytron. The samples were shipped to Asuragen Services for microRNA profiling. Samples for microRNA profiling studies were processed by Asuragen Services according to the company’s standard operating procedures. Briefly, a custom-manufactured Affymetrix GeneChip from Ambion was designed to microRNA probes derived from Sanger mirBase 9.2 and published reports [1, 2, 3, 4]. The array was designed with 2 staggered probes to represent each microRNA. The signal processing implemented for the Ambion miRCHIP is a multi-step process involving probe specific signal detection calls, background estimate and correction, constant variance stabilization [5], and either array scaling or global normalization. For each probe, an estimated background value is subtracted that is derived from the median signal of a set of G-C-matched anti-genomic controls. Arrays within a specific analysis experiment were normalized together according to the variance stabilization method described by Huber et al. [5]. Detection calls were based on a Wilcoxon rank-sum test of the microRNA probe signal compared to the distribution of signals from GC-content matched anti-genomic probes. For statistical hypothesis testing, a two-sample t-Test with assumption of equal variance was applied. One-way ANOVA was used for experimental designs with more than two experimental groupings or levels of the same factor. These tests define which probes are considered to be significantly differentially expressed, or significant, based on a default p-value of 0.001 and log2 difference > 1.

**TaqMan validation of microarray results for normal and xenograft for miR-21, miR-182, miR-221 and miR-222.** Absolute copy number of mature microRNAs was determined by quantitative real-time PCR (qRT-PCR) using Assays-on-Demand Taqman primer/probe sets along with Taqman Universal PCR master mix (Applied Biosystems Inc., Foster City, CA) for cDNA amplification. Amplification and analysis were performed on the ABI 7000 sequence detection system. Copies per cell were determined using an estimated 15 pg total RNA per cell.

# References

1. Cummins JM, He Y, Leary RJ, Pagliarini R, Diaz LA,Jr, Sjoblom T, Barad O, Bentwich Z, Szafranska AE, Labourier E, Raymond CK, Roberts BS, Juhl H, Kinzler KW, Vogelstein B, Velculescu VE : **The colorectal microRNAome.** *Proc Natl Acad Sci U S A* 2006, **103:**3687-3692.

2. Bentwich I, Avniel A, Karov Y, Aharonov R, Gilad S, Barad O, Barzilai A, Einat P, Einav U, Meiri E, Sharon E, Spector Y, Bentwich Z : **Identification of hundreds of conserved and nonconserved human microRNAs.** *Nat Genet* 2005, **37:**766-770.

3. Berezikov E, Guryev V, van de Belt J, Wienholds E, Plasterk RH, Cuppen E : **Phylogenetic shadowing and computational identification of human microRNA genes.** *Cell* 2005, **120:**21-24.

4. Xie X, Lu J, Kulbokas EJ, Golub TR, Mootha V, Lindblad-Toh K, Lander ES, Kellis M : **Systematic discovery of regulatory motifs in human promoters and 3' UTRs by comparison of several mammals.** *Nature* 2005, **434:**338-345.

5. Huber W, von Heydebreck A, Sultmann H, Poustka A, Vingron M : **Variance stabilization applied to microarray data calibration and to the quantification of differential expression.** *Bioinformatics* 2002, **18 Suppl 1:**S96-104.
