# Supplementary figures and images for: Molecular assays for the detection of microRNAs in prostate cancer
Source: Mol Cancer. 2009 Mar 6;8:17. doi: 10.1186/1476-4598-8-17 (PMC2656451; doi:10.1186/1476-4598-8-17)

**Figure S2.**


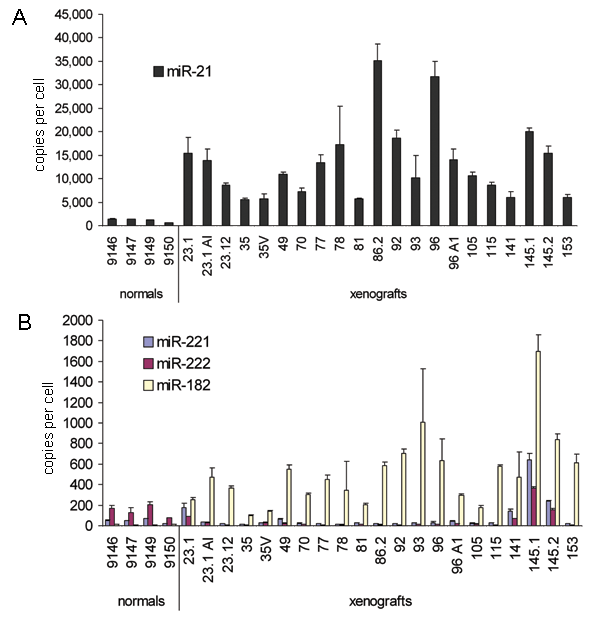

Supplement: Additional file 4 — Validation of miR-21, miR-182, miR-221 and miR-222 expression levels in human adjacent-normal and prostate tumor xenograft tissues using a commercial quantitative RT-PCR assay. Bar graphs of copy numbers per cell determined using a commercial quantitative RT-PCR assay. Synthetic miRNAs were diluted 10-fold serially from 109 copies per reaction for construction of each standard curve. For each reverse transcription reaction, 10 ng of total RNA was used and amplification reactions were run in triplicate (see Additional file 1 for details). [file 1476-4598-8-17-S4.doc]
